# Post-traumatic stress disorder and stroke in the elderly

**DOI:** 10.1192/j.eurpsy.2021.1206

**Published:** 2021-08-13

**Authors:** M. Chakroun, R. Ouali, O. Zribi, M. Turki, L. Aribi, J. Aloulou

**Affiliations:** 1 Psychiatry (b), Hedi Chaker University hospital, sfax, Tunisia; 2 Psychiatry B Chu Hedi Chaker, Tunisia, psychiatry B, sfax, Tunisia; 3 Psychiatry (b), Psychiatry (B), Hedi Chaker University hospital, sfax, Tunisia; 4 Psychiatry (b), Hedi Chaker University hospital, Sfax, Tunisia

**Keywords:** PSTD, stroke, Elderly

## Abstract

**Introduction:**

Posttraumatic stress disorder (PTSD) is common in survivors of acute life-threatening illness, but little is known about the burden of PTSD in survivors of stroke attack.

**Objectives:**

This study estimated the prevalence of PTSD in post-stroke in the elderly and to look for the factors which are correlated with it.

**Methods:**

Participants were outpatients of Psychiatry B department in Hedi chaker University Hospital Center in Tunisia, over the age of 65, hospitalized in psychiatry for a major depressive episode, recruted between 2000 and 2015. The data was collected using a pre-established sheet containing socio-demographic information, the clinical and evolutionary characteristics of the depressive episode and the therapeutic data concerning the depressive episode.

**Results:**

30 patients were included in this study with an average age (69 Y) and sex ratio (0.66). More than half (53.3%, 16 patients) had a history of chronic somatic disease. The average length of hospitalization was 26 days. The most frequent reason for hospitalization is sadness of mood (43.3%) with cognitive impairment as the predominant clinical symptomatology (40%). 93.3% of the population received as treatment an antidepressant mainly Fluoxetine (50%).

**Conclusions:**

clinicians should be mindful that PTSD can be a devastating mental health condition and should consider screening for PTSD in stroke survivors.

